# Enhancing Abiotic Stress Resilience in Mediterranean Woody Perennial Fruit Crops: Genetic, Epigenetic, and Microbial Molecular Perspectives in the Face of Climate Change

**DOI:** 10.3390/ijms26073160

**Published:** 2025-03-29

**Authors:** Aliki Kapazoglou, Eleni Tani, Vasileios Papasotiropoulos, Sophia Letsiou, Maria Gerakari, Eleni Abraham, Penelope J. Bebeli

**Affiliations:** 1 Department of Grapevine, Institute of Olive Tree, Subtropical Crops and Viticulture (IOSV), Hellenic Agricultural Organization-Dimitra (ELGO-Dimitra), Lykovrysi, 14123 Athens, Greece; 2 Laboratory of Plant Breeding and Biometry, Department of Crop Science, Agricultural University of Athens, Iera Odos 75, 11855 Athens, Greece; etani@aua.gr (E.T.); vpapasot@aua.gr (V.P.); mgerakari@aua.gr (M.G.); bebeli@aua.gr (P.J.B.); 3 Department of Food Science and Technology, University of West Attica, Egaleo, 12243 Athens, Greece; sletsiou@uniwa.gr; 4 School of Forestry and Natural Environment, Aristotle University of Thessaloniki, Thessaloniki, 54124 Thessaloniki, Greece; eabraham@for.auth.gr

**Keywords:** tolerance, drought, salinity, microbiome, epigenome

## Abstract

Enhanced abiotic stresses such as increased drought, elevated temperatures, salinity, and extreme weather phenomena severely affect major crops in the Mediterranean area, a ‘hot spot’ of climate change. Plants have evolved mechanisms to face stressful conditions and adapt to increased environmental pressures. Intricate molecular processes involving genetic and epigenetic factors and plant–microbe interactions have been implicated in the response and tolerance to abiotic stress. Deciphering the molecular mechanisms whereby plants perceive and respond to stress is crucial for developing strategies to counteract climate challenges. Progress in determining genes, complex gene networks, and biochemical pathways, as well as plant–microbiota crosstalk, involved in abiotic stress tolerance has been achieved through the application of molecular tools in diverse genetic resources. This knowledge could be particularly useful for accelerating plant improvement and generating resilient varieties, especially concerning woody perennial crops, where classical breeding is a lengthy and labor-intensive process. Similarly, understanding the mechanisms of plant–microbe interactions could provide insights into innovative approaches to facing stressful conditions. In this review, we provide a comprehensive overview and discuss the recent findings concerning the genetic, epigenetic, and microbial aspects shaping abiotic stress responses, in the context of enhancing resilience in important Mediterranean woody perennial fruit crops.

## 1. Introduction

In upcoming decades, the Mediterranean region will be significantly impacted by climate change, based on the model predictions of the Intergovernmental Panel on Climate Change (IPCC)’s Sixth Assessment Report [1]. Depending on greenhouse gas emissions, rainfall is predicted to decrease by 4–22%, whereas the intensity and frequency of extreme weather events such as drought spells and heat waves, as well as erratic precipitation patterns and storm surges in certain areas, are also anticipated to increase. These alarming forecasts call for concerted actions by agronomists, farm practitioners, and researchers to mitigate the severe effects of unfavorable environmental conditions on crop production and agricultural sustainability [2,3,4].

Perennial woody fruit crops play a crucial role in the agriculture of the Mediterranean region. However, their productivity remains below their potential due to various biotic and abiotic challenges associated with the ongoing climate change. Woody crops are highly vulnerable to climate impacts due to their long cultivation period, dependence on rain-fed cultivation, and the substantial upfront costs that make crop rotation challenging. As a result, the sector requires well-designed adaptation strategies to cope with climate threats that may include transformative change. Establishing perennial cropping systems demands significant capital investment and an extended timeframe, making adaptation strategies such as crop switching far less feasible compared to annual crops [5]. Importantly, there remains a significant knowledge gap regarding how climate change differentially impacts perennial versus annual cropping systems.

In this context, perennial plants such as fruit trees undergo a dormancy phase, during which physiological activity slows down, primarily in response to temperature and, in some species, to day length [6]. Breaking dormancy typically requires both chilling and heating accumulations, making these crops particularly sensitive to shifts in seasonal temperatures. A decline in winter chill has already been observed in temperate and subtropical regions, and this trend is expected to continue in the future. Insufficient chilling hours can delay bloom or leaf emergence, although some delays may be mitigated by faster heat accumulation in spring [6,7]. These changes pose significant challenges for fruit tree growers, who may need to replace existing species or cultivars with those better suited to future climatic conditions [8]. Nevertheless, knowledge is still obscure with respect to the heat and chill requirements of different perennial crop species and cultivars, the ability to model these requirements accurately, and the dormancy phases relevant to heat and chilling hour accumulation. Addressing this knowledge gap is crucial for developing effective adaptation strategies and ensuring the long-term viability of perennial cropping systems under changing climatic conditions. Delineating the molecular mechanisms regulating these processes will be of particular importance, as it will provide valuable tools for designing successful breeding programs. Plant breeding represents one of the primary strategies to tackle the problems caused by climate change and secure the production and sustainability of perennial crops under adverse conditions [9]. Conventional breeding primarily utilizes phenotypic data [10] but has several limitations, including the lengthy process (over 10 years) required to develop a new variety and the influence of environmental factors, which lowers heritability for many traits, especially complex ones like yield. On the other hand, molecular plant breeding can greatly increase the speed, efficiency, and precision of breeding compared to conventional methods [11].

**Figure 1:**
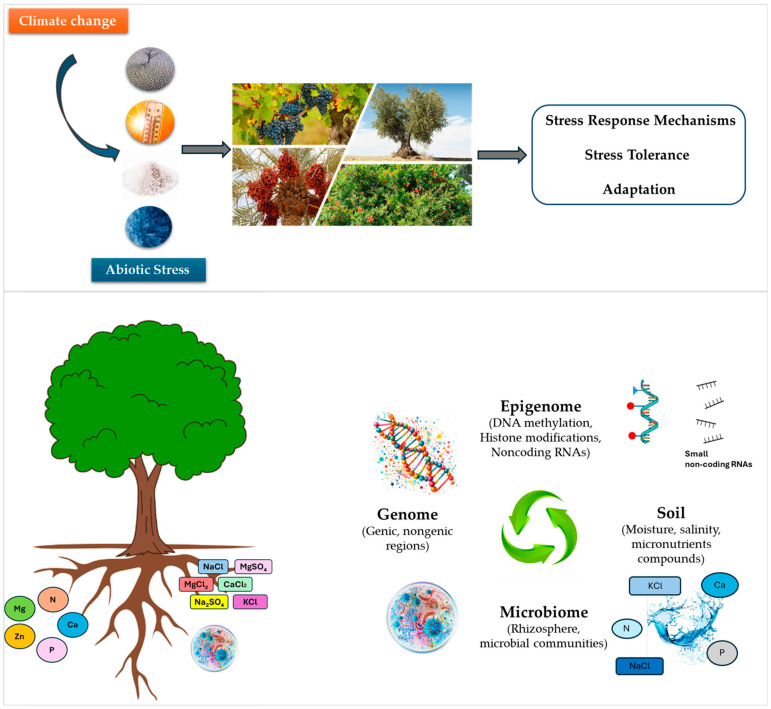
A schematic view of the factors influencing the response to abiotic stresses and plant–microbe interactions in woody fruit species. Abiotic stresses such as drought, temperature, salinity, and cold induce stress response mechanisms that lead to plant tolerance and adaptation. Soil properties, water and nutrient availability, and the synergies of the genome, the epigenome, and the microbiome are crucial elements for proper plant growth and stress resilience under adverse conditions. Beneficial microorganisms such as rhizosphere microbial communities are involved in many physiological and molecular processes and can promote plant growth, increase water and nutrient uptake, and enhance resilience to environmental stresses. These activities depend on genetic, epigenetic, and microbial factors and soil physicochemical properties and entail intricate gene networks and epigenetic regulation such as DNA methylation (red circles), histone modifications (blue triangles), and the action of small noncoding RNAs modulating gene expression dynamics.

Next-generation sequencing and novel molecular approaches like genomics, epigenomics, transcriptomics, proteomics, metabolomics, genome-wide association studies (GWASs), and genome editing (e.g., CRISPR/Cas) have advanced the study of abiotic stress tolerance in plants. When applied to diverse genetic resources, these tools help uncover stress-related gene networks and metabolic pathways, enabling the development of superior, stress-tolerant genotypes through genomic selection and modern breeding [12,13,14,15,16,17,18,19,20,21]. Special attention is given to genomic prediction, which estimates marker effects by analyzing all genotyped markers simultaneously, capturing more phenotypic variations than GWASs and accurately dissecting genetic variance. In tree breeding, genomic prediction enables the early identification of superior plants in the seedling stage, bypassing long maturation periods [22]. This accelerates breeding efficiency and genetic gains [23,24]. However, there are several drawbacks that slow the breeding of perennial crops such as their long juvenile phase (5–9+ years) and the lengthy backcross breeding process required. To avoid missing opportunities in genomics-assisted breeding and genetic engineering (GE)—already transforming cereals and legumes—the adoption of new breeding technologies is imperative [25]. Therefore, understanding the cellular and molecular mechanisms behind abiotic stress perception, response, and tolerance is crucial for accelerating woody perennial fruit crop improvement. These processes involve complex gene networks and expression dynamics triggered by various environmental challenges. Furthermore, epigenetic factors, such as DNA methylation, histone modifications, and the action of noncoding RNAs, play crucial roles in regulating the molecular mechanisms associated with the abiotic stress response and the establishment of tolerance [12,26,27] (Figure 1). Although many studies are available on epigenetic regulation in abiotic stress responsiveness (such as DNA methylation changes caused by drought stress) in annual, herbaceous plants, few reports are available for perennial woody plants focusing mainly on poplar and apple trees [28,29], underscoring the need for further investigations in woody perennial species [30].

Moreover, multiple and ever-increasing studies have highlighted the vital role of the microbiome (soil, rhizosphere, phyllosphere, carposphere) on plant development and environmental stress resilience [31,32,33]. Plant-associated microbiota could be either pathogenic or beneficial. Microbiota include various classes of microorganisms, such as epiphytes, endophytes, and arbuscular mycorrhiza. It is well recognized that these microorganisms have the capacity to exert beneficial effects on plants by promoting growth, increasing water and nutrient uptake, and enhancing resilience to environmental stresses. Numerous studies have shown that both the host plant and beneficial microorganisms are able to confer abiotic stress tolerance through a wide range of physiological and molecular mechanisms [13,31,34,35,36,37].

Considering the re-emerging notion of the plant as an ‘holobiont’, which refers to the assemblage of the host plant along with its associated microbiota, our view of the plant’s responses has expanded to include the microbiome of the epiphytic and endophytic microbiota (e.g., bacteria, fungi, viruses) associated with the plant’s organs and adjacent soil [38,39,40]. A wealth of investigations, mainly in annual herbaceous plants, has explored the genetic and environmental drivers of microbiota diversity, the structure of microbial communities, and host–microbiota associations, both belowground and aboveground (soil, rhizosphere, phyllosphere, carposphere), as well as the interactions within these complex systems [32,33,34,35,36,41,42,43,44,45] (Figure 1).

Over the last several years, research efforts have begun to unravel the molecular dynamics of the abiotic stress response in woody perennial species. In the current review, we present a comprehensive survey of the progress achieved regarding the genetic, epigenetic, and microbial facets of abiotic stress responsiveness and tolerance in key woody fruit crops of the Mediterranean basin. Furthermore, we discuss the significance of these multidimensional aspects in successfully designing innovative strategies for mitigating the negative impact of global climate change on woody fruit crop sustainability.

## 2. Grapevine

### 2.1. Climate Challenges

Grapevine (*Vitis vinifera* L.) is a woody perennial plant belonging to the genus *Vitis,* which harbors 60–80 species (*Vitis* spp.). Grapevine domestication dates back to 11,000–8000 B.C., and it has been estimated that the species *Vitis vinifera* encompasses 6000–10,000 cultivars with very high inter- and intra-varietal genetic diversity [46,47,48]. Nevertheless, only a portion of this wide genetic pool has been exploited for commercial use [17,48,49].

Cultivated grapevine constitutes a crop of high economic value, intimately linked to the history and cultural heritage of viticultural regions around the world [48]. The global vineyard surface area has expanded to approximately 7.2 million hectares (mha) with an annual grape production of ~77.8 million tons, of which 47.4%, 44.5%, and 8% correspond to wine, table, and dried grape, respectively [50]. It has been well acknowledged that mild drought stress is beneficial for winegrape varieties as it promotes higher sugar content and lower acidity as well as the synthesis of metabolites (polyphenols, tannins, volatile organic compounds (VOCs)), determining color, aroma, and flavor, all leading to improved wine quality [51]. However, in recent years, grapevine cultivation has witnessed severe environmental pressures, characterized by prolonged drought periods, escalating temperatures, and enhanced rainfall, which pose major threats both for wine and table grape productivity and the sustainability of viticulture [48,52]. Moreover, the increased salinization of agricultural lands in grape-growing regions reduces grapevine production and raises major concerns for the future of viticulture.

In 2023, global wine production declined by 10% as compared to 2022, whereas a further decline is anticipated for 2024 [50]. Extreme weather phenomena including early frosts, protracted drought, and heavy rainfall leading to the exacerbation of fungal diseases have dramatically impacted vineyard harvests worldwide. To address adverse environmental changes and dire climate forecasts, viticulturists, producers, the wine industry, and researchers have adopted various strategies such as (a) improving vineyard management and implementing effective cultivation practices adapted to current challenges [52,53,54]; (b) capitalizing on grapevine’s great genetic diversity and exploring the wide allele pool of *Vitis* spp. to develop superior genotypes with enhanced stress resilience [17,19,49]; and (c) exploiting the growing knowledge on the grapevine microbiome, especially the capacity of rhizosphere microbiota to confer stress tolerance, an approach gaining significant attention lately [35,55,56]. An important aspect across all strategies is the effective use of rootstocks to optimize scion performance. The ancient practice of grafting scions onto a suitable rootstock significantly improves plant qualities as it imparts the plant with advantageous adaptive traits. Historically, European grapevine has been grafted onto interspecific North American hybrid rootstocks to counteract the catastrophic infestations of phylloxera (*Daktulosphaira vitifoliae*) [57,58]. Additionally, cultivated grapevine varieties are grafted onto compatible rootstocks to ensure productivity, fruit quality, and resistance to disease [49,57,59]. Certain rootstocks have the potential to provide abiotic stress tolerance, whereas successful graft performance relies on the compatibility of rootstock and scion genotypes [49,57,59,60,61]. Consequently, the proper management of rootstocks represents a sound strategy to augment the resilience of grapevines to unfavorable environmental conditions. Nevertheless, the genetic basis of grapevine rootstocks is quite narrow, and improvements in existing or novel varieties have become necessary. Equally, appropriate and improved scion varieties are sought to satisfy the enhanced resilience requirements. Thus, another effective approach to mitigate the negative effects of abiotic stresses is the implementation of technology to develop abiotic-stress-tolerant grapevine cultivars. In this regard, delineating the genetic and epigenetic mechanisms underlying abiotic stress responsiveness and rootstock–scion crosstalk will be crucial for crop improvement in both grafting partners [61]. Similarly, comprehending microbiome structure/function and harnessing the potential of the grapevine microbial communities to confer tolerance to abiotic stresses that damage grapevine productivity could lead to alternative protection approaches [49,62,63].

### 2.2. Genetic and Epigenetic Attributes of Abiotic Stress

Elucidating the molecular mechanisms of tolerance to abiotic stresses and implementing this knowledge to crop improvement rely intensely on studying the genetic and epigenetic components shaping grapevine performance (Table 1).

The high genetic variability and rich allelic reservoir characterizing *Vitis* spp. constitute valuable resources for understanding stress response mechanisms and designing improvement strategies toward enhanced tolerance to abiotic stresses [17,49]. A plethora of genetic studies have identified SSR and SNP markers related to specific grapevine varieties and important agronomic characteristics, thereby providing molecular means that could substantially aid the selection of desired genotypes in breeding programs [64,65,66,67,68,69,70,71,72].

Moreover, the advancement in DNA sequencing technologies has led to the release of improved versions of grapevine reference genome assemblies [73] as well as a non-reference pangenome of wild grapevine accessions [74]. These genomic resources serve as a valuable tool to understand the molecular basis of morphological, physiological, and biochemical characteristics related to yield, fruit quality, and stress resilience. Importantly, the recent construction of a grapevine pangenome reference map [75], encompassing resequencing data from 466 grapevine cultivars and including a remarkable range of short variants as well as structural variants (SVs), is anticipated to decipher complex polygenic trait associations and contribute to genomic breeding [59,60].

Recently, Genome-Wide Association Studies (GWASs) have been conducted to dissect the genetic basis of abiotic-stress-tolerance-related traits in grapevine [17,49]. GWASs were used to explore stomatal conductance during water deficit stress utilizing 100 distinct grapevine genotypes, which included interspecific hybrids and various rootstock varieties. Genomic regions encompassing 24 significant SNP marker–trait associations with relevance to drought tolerance were detected, and thirteen candidate genes possibly responsive to water deficit were identified [65]. Notably, one of these candidate genes encoded a raffinose synthase, indicating a role for this enzyme in the early response to drought stress and agreeing with the known protective function of raffinose oligosaccharides against abiotic stressors [76]. Moreover, the whole-genome re-sequencing of 77 rootstock genotypes (including North American and Asian *Vitis* species and their hybrids) and GWAS analysis revealed six sets of 631, 13, 9, 2, 810, and 44 SNPs, with substantial relevance to resistance/tolerance to phylloxera, root-knot nematodes, salt, drought, cold, and waterlogging, respectively [77].

Transcriptomic profiling based on genome-wide RNA sequencing has highlighted the important aspects of gene expression programs and the molecular mechanisms associated with abiotic stress responsiveness. A study of drought-tolerant and drought-susceptible grapevine rootstocks revealed the differential expression of genes encoding the transcription factors VvAGL15, VvBD41, and VvMYB86 and a range of proteins implicated in antioxidant pathways, suggesting their putative roles in the regulation of the molecular processes underlying drought response and tolerance [78]. Investigation of the effects of combined drought and heat stress at the physiological and molecular level revealed the co-expression of gene networks encompassing signal transduction cascades, phenyl propanoid metabolism, sugar-metabolizing enzymes, heat-shock protein and transcription factor regulation, and histone modification factors. Notably, most of the differentially expressed genes were unique to the individual or combined stress types, and only a few were shared among the two, pointing to the operation of distinct mechanisms [79]. Moreover, transcriptomic analysis of grapevine genotypes with variable responses to heat stress showed the differential regulation of genes encoding transcription factor families of the types HSP, WRKYs, MYBs, and NACs, or regulators involved in auxin and ABA signaling and starch and sucrose metabolic networks, highlighting the importance of these key pathways in the response to high-temperature stress [80].

Concerning epigenetic regulation and transcriptional reprogramming during grapevine development and stress response, several investigations have indicated altered cytosine methylation patterns, differential histone modifications, and the action of diverse classes of noncoding RNAs (miRNAs and siRNAs) in response to drought, temperature, salt, and UV-B radiation stresses [54,61,81,82]. More specifically, Pagliarani et al. reported that water deficit conditions induced significant downregulation of the well-conserved microRNA 159, *miR159*, in a drought-tolerant hybrid (M4) but not in a drought-sensitive variety (Cabernet Sauvignon) under both greenhouse and field conditions [83]. In addition, the transcript abundance of the conserved microRNA156 miR156 was reduced upon drought stress in both genotypes. Conversely, a microRNAome study demonstrated the upregulation of miRNAs *miR159c*, *miR156b,* and *miR156f* under drought conditions in the susceptible genotype (Cabernet Sauvignon) but not in the tolerant genotype (110 Richter rootstock) [84]. Recent investigations on the response to drought of indigenous grapevine varieties from traditional viticultural regions identified substantial variability in drought tolerance among distinct genotypes, accompanied by differential miRNA regulation [85]. *miRNA159* displayed a marked induction in the drought-tolerant variety ‘Dichali’, both in self-rooted plants and those grafted on the 110R rootstock, but not in the drought-sensitive variety ‘Debina’. On the other hand, *miR156* showed a significant induction only in severely stressed self-rooted plants of the drought-tolerant ‘Dichali’ [85]. These findings suggest that the expression patterns of *miRNA159* and *miR156* are associated with grapevine drought responsiveness and tolerance in a genotype- and grafting-dependent manner. Furthermore, miRNA up- or downregulation was linked to the anticorrelated expression patterns of putative target genes, *MYB1* and *TRP*, encoding key transcription factors involved in development and stress response processes [83,85]. These results suggested the involvement of ‘*miRNA-Transcription factor*’ regulatory modules characterizing the drought response and are in line with previous studies in grapevine and other plant species [86,87,88]. Furthermore, other investigations have shown the differential expression of large sets of miRNAs under salinity or cold stress and indicated miRNA subgroups as regulators of key downstream genes associated with stress responsiveness [89,90]. Studies on DNA methylation and histone remodeling have begun to emerge. The treatment of Cabernet Sauvignon seedlings with 5-azacytidine and the subsequent decrease in global cytosine methylation led to an effective reduction in aluminum toxicity, presumably by upregulating stress-responsive genes [78,91]. Additionally, the histone modification profiling of *V. amurensis* leaves demonstrated massive genome-wide H3K27 trimethylation modifications upon chilling stress, associated with various pathways including stress-responsive gene networks [92].

Collectively, the findings described above underscore the important role of genetic and epigenetic regulation in abiotic stress responses and unveil the multiple factors that may shape stress tolerance (genotype, grafting, developmental stage, type of stress). A deeper comprehension of the molecular mechanisms underlying key responses to abiotic stressors, engaging both scions and rootstocks, will expand our knowledge of grapevine stress tolerance. Ultimately, the research outcomes will be translated into practical application by developing abiotic-stress-tolerant varieties in breeding programs. Along these lines, it will enable progress in the effective implementation of genome editing approaches for accelerated grapevine improvement (e.g., CRISPR-Cas) [93]. To date, an increasing number of studies have been reported on grapevine genome editing, mostly focusing on the enhancement in biotic stress resistance, carotenoid biosynthesis, and sugar and flavonoid accumulation. Despite challenges related to transformation and regeneration efficiencies depending on genotype, these outcomes are encouraging, and it is anticipated that progress will be achieved in the domain of abiotic stress tolerance, paving the way to climate-resilient viticulture.

**Table 1:** Abiotic stress responses associated with genomics and epigenomics.

Species	Stress Type	Molecular Tools	Molecular Response/Tolerance-Associated Genes	References
**Grapevine**	Drought	Genome-wide association studies (GWASs)	Candidate genes and SNPs associated with stomatal conductance and drought responsiveness, e.g., raffinose synthase.	[65,77]
		Transcriptomics-RNA Seq/Quantitative PCR	Co-expression of gene networks related to signal transduction cascades, phenyl propanoid metabolism, sugar-metabolizing enzymes, heat-shock protein transcription factor regulation, and histone modification factors.	[79]
			TF families VvAGL15, VvLBD41, and VvMYB86	[78]
			Up- and downregulation of responsive miRNAs VvmiR159 and VvmiR156.	[83]
			Induction of miRNAs VvmiR159 and VvmiR156 and anticorrelated expression of TF genes MYB1 and TPR.	[85]
			Drought-induced VvmiR169d and VvmiR156b upregulation and VvmiR398a downregulation.	[84]
			Activation of the module miR156b-VvSBP8/13.	[87]
	Heat	Transcriptomics-RNA seq/Quantitative PCR	Transcription factor families WRKYs, MYBs, and NACs; auxin and ABA signaling; starch and sucrose metabolism.	[80]
			Induction of heat-stress-responsive miRNA VvmiR167.	[71]
	Aluminum (Al) toxicity	Whole-genome bisulfite sequencing (WGBS)	DNA methylation reduction/enhanced tolerance to Al.	[91]
	Cold	Chromatin immunoprecipitation (ChIP) Transcriptomics-RNA seq	H3K27 trimethylation alterations/gene target downregulation.	[92]
			Novel cold-stress-responsive microRNAs.	[90]
**Olive tree**	Drought	Transcriptomics/RNA-seq	Transmembrane transport and metal ion binding processes, and abscisic acid, gibberellin, brassinosteroid, and ethylene-activated signaling.	[94]
	Salt	Transcriptomics/RNA-seq	TF families JERF and bZIP.	[95]
			Upregulation of OeNHX7, OeP5CS, OeRD19A, and OePetD.	[96]
**Date Palm**	Combined heat and drought	Proteomics	Increased abundance of heat shock proteins (HSPs), redox homeostasis proteins, and proteins involved in isoprene production.	[97]
	Salt	Multi-omics	Converging gene expression and protein abundance associated with osmotic adjustment, reactive oxygen species scavenging in leaves, and remodeling of the ribosome-associated proteome in salt-exposed root cells.	[98]
			Induction of salt overly sensitive (SOS) genes PdSOS2;1, PdSOS2;2, PdSOS4, PdSOS5, and PdCIPK11.	[99]
		Whole-genome bisulfite sequencing (WGBS)	Differential DNA methylation and gene expression alterations in the roots.	[100]
**Pomegranate**	Salt	Transcriptomics/RNA-seq	Spatiotemporal regulation of SWEET genes.	[101]
			DEGs associated with ABA- and Ca^2+^-related and MAPK signal transduction pathways (ABA-receptors, Ca^2+^-sensors, MAPK cascades, TFs) and downstream functional genes coding for HSPs, LEAs, AQPs, and PODs.	[102]
			Induction of proline, total soluble sugar, and SOD/POD activities and differential gene expression.	[103]
	Cold	Transcriptomics/RNA-seq	Upregulation of CBF genes PgCBF3 and PgCBF7.	[104]
			Differentially expressed genes related to TFs, photosynthesis, the osmotic regulation system, hormone signal transduction, and sucrose metabolism.	[105]
			Induction of beta-amylase, PgBAM4, and increase in soluble sugar content.	[106]

### 2.3. Microbiota Attributes Related to Abiotic Stress

Concerning the microbiome aspect as it pertains to abiotic stress, several studies have shown that grapevine microbiota are capable of conferring resilience to abiotic stress factors such as drought, elevated temperature, and high salinity by activating plant growth mechanisms, increasing photosynthetic capacity, synthesizing phytohormones (e.g., IAA, ABA), accumulating osmoprotectants (e.g., proline, trehalose) and ROS scavenging antioxidant molecules, inducing stress-responsive genes, and triggering other cell-protecting mechanisms [55,56] (Table 2).

Earlier laboratory and greenhouse studies identified plant growth-promoting (PGP) bacterial strains capable of colonizing the grapevine rhizoplane and supporting plant growth under water-deficient conditions by increasing shoot/leaf biomass and shoot length, inducing photosynthetic activity, and accumulating hormones as well as producing defense-related products such as terpenes [107,108]. Importantly, bacterial subsets originating from the grapevine roots and rhizosphere were found to exert beneficial effects on field-grown grapevines in various biogeographical settings [109].

In addition, mycorrhizal and endophytic fungi have also been shown to alleviate drought, temperature, and salt stress through a variety of mechanisms such as modulating ABA and auxin metabolism and inducing antioxidant activity [55,110]. Recently, arbuscular mycorrhizal fungus (AMF) inoculation of a *Vitis vinifera* L. cv. Ecolly was demonstrated to confer drought tolerance by increasing the accumulation of osmolytes, triggering antioxidant processes, and regulating the expression of key stress-responsive genes *VvNCED*, *VvP5CS*, *VvSIP*, *VvPIP1;2*, and *VvTIP2;1* [111]. Moreover, field experiments conducted in periods of rising temperatures and reduced water availability showed that grapevine rootstocks inoculated with AMF displayed enhanced growth and survival compared with non-AMF-associated plants [112]. In another study, growth-promoting rhizobacterium consortia from the marine plant significantly improved the resilience of grapevine to heat stress, highlighting the potential of microbial consortia to promote fitness and protect grapevine from frequent heatwave exposure [113,114].

Notably, global biogeography surveys have linked microbiomes with entire viticultural regions. Large-scale studies of vineyard rhizosphere microbiomes across continents revealed remarkable associations among microbiomes, terroir, and wine characteristics [115,116]. Microbiome ‘’signatures’’ could be ascribed to distinct vineyards within and between continents and countries, highlighting the microbiome’s potential as a fingerprinting tool to trace the geographical origin of grapevine germplasm and ensure the authenticity of end products. Considering that terroir-associated microbiomes are shaped by rootstock/scion genotype, soil, and climate factors [63,117,118], it is conceivable that microbiota consortia from hot and dry areas may confer tolerance to grapevines from more temperate zones currently undergoing temperature and drought pressures [35,63].

Finally, concerning the mechanistic complexities of plant–microbe interactions, a major challenge will be to decode the intricate molecular interplay between the genetic, epigenetic, and microbial components driving the response to abiotic stress. In this respect, little progress has been made and involves a few annual herbaceous plants like Arabidopsis, chickpea, and herbs [44]. For example, it was shown that *Pseudomonas putida* (a plant growth-promoting rhizobacterium (PGPR)), known to improve performance under abiotic stress, mediates the regulation of specific chickpea (*Cicer arietinum* L.) miRNAs and their targets in response to drought and salt stress [119]. Interestingly, the *Pseudomonas putida*-induced microRNA, *car*-miR166, was found to play a stress-mitigating role in PGPR-inoculated Arabidopsis transgenic lines under drought conditions [114].

Furthermore, Pokeweed (*Phytolacca americana* L.), a North American native perennial herb with great potential for bioremediation of heavy-metal-contaminated soils, displayed altered DNA methylation in roots upon inoculation with PGPR. Importantly, the DNA methylation modification was associated with enhanced plant growth and persisted even after removal of the inoculum [120].

Recently, investigations have emerged aiming to explore the molecular dynamics of the ‘grapevine–microbiota–stress’ interactions. Campos et al. studied *V. vinifera* cv. Touriga Nacional grafted onto 1103 Paulsen rootstocks, inoculated with AMF, and subjected to high-temperature stress. The AMF inoculation of grapevine roots enhanced the physiological indices under temperature stress. Notably, it also up- or downregulated specific miRNAs that target genes encoding stress-related transcription factors or proteins involved in antioxidant pathways, suggesting that mycorrhiza-mediated miRNA regulatory networks act in response to heat stress [121]. Furthermore, the variation in miRNA expression was observed in leaf tissue, pointing to molecular signaling control between the rootstock and scion by mycorrhization and stress factors. Interestingly, in another study, a comparative transcriptome analysis of ten grapevine rootstocks revealed the induction of common as well as unique sets of genes after mycorrhiza inoculation, indicating genotype-specific gene expression upon mycorrhization [122] that may have stress-response relevance. These findings set the foundation for further investigations of the complex molecular interplay between the grapevine plant and the associated microbiota under abiotic stress conditions.

A deeper understanding of the response to abiotic stress, the role of the genetic and epigenetic factors involved, and the grapevine–microbe interplay will illuminate the molecular processes involved in abiotic stress responsiveness and tolerance and lead to innovative approaches for climate-smart viticulture.

**Table 2:** Abiotic stress responses associated with microbiomes.

Species	Stress Type	Microbe Type	Microbial Effect—Molecular Response	References
**Grapevine**	Drought	Rhizosphere-associated bacteria	Protection against reactive oxygen species (ROS)—accumulation of terpenes.	[108]
	Drought	Root-associated microbiome	Water stress protection.	[107]
	Drought	Arbuscular mycorrhizal fungi (AMF)	Drought tolerance by increasing the accumulation of osmolytes, triggering antioxidant processes, and regulating the expression of key stress-responsive genes.	[111]
	Heat	Marine plant growth-promoting rhizobacteria consortia	Heat stress tolerance.	[113]
	Heat	AMF	Enhancement in physiological indices; modulation of miRNAs and stress-related transcription factors and proteins related to antioxidant pathways.	[121]
**Olive tree**	Drought	*Pseudomonas reactans* Ph3R3	Enhancement in plant performance by reducing water loss and improving N levels, net CO_2_ assimilation rate, and antioxidant capacity.	[123]
	Drought	PGPB consortia sampled from the soil and rhizosphere of Tunisian olive orchards	Conferred tolerance to both drought-susceptible and drought-tolerant cultivars.	[124]
	Drought	AMF (*Rhizophagus irregularis*)	Reinforced tolerance to water deficit by enhancing olive plant growth and improving water status, accumulation of osmolytes and antioxidants, and phytohormone regulation.	[125]
	Drought	AMF (*Rhizophagus irregularis*)	Enhanced water deficit tolerance by increasing net carbon fixation, water use efficiency, and antioxidant defenses.	[126]
	Salt	PGPB *Bacillus G7*	Improved physiological and metabolic parameters, and increased photosynthetic capacity, net carbon fixation, water use efficiency, and accumulation of osmolytes and antioxidants.	[121]
	Salt	AMF mixture of *Glomus deserticola* and *Gigaspora margarita*	Alleviation of the stress imposed by irrigation with salt-enriched wastewater.	[127]
**Date Palm**	Drought	Selected date plam root bacterial endophytes	Increased the biomass of date palms exposed to recurrent drought stress cycles in a greenhouse experiment.	[128]
	Salt	*Piriformospora indica* endophyte	Mitigated the detrimental effects of salt stress through ion homeostasis and nutrient uptake, antioxidant activity, and upregulation of stress-responsive genes.	[129,130]
	Salt	*Enterobacter cloacae* SQU-2 (SQU-2)’	Improved the growth of *Arabidopsis thaliana* Columbia (Col-0) seedlings under both normal and salt stress conditions through the production of microbial volatile compounds (mVOCs).	[131]
**Pomegranate**	Drought	AMF strains *Rhizophagus intraradices* (GA5 and GC2)	Early inoculation with AMF, especially for the GC2 strain, offers protection against drought; antioxidant defenses, specifically the ROS-scavenging enzymes superoxide dismutase (SOD), catalase (CAT), and ascorbate peroxidase (APX), are enhanced in shoots.	[132]

## 3. Olive Tree

### 3.1. Climate Challenges

Olive belongs to the botanical family Oleaceae, harboring about 30 genera and 600 species. The Olea L. genus comprises 33 species, with *Olea europaea* L. being the only cultivated species with more than 2000 cultivars [133]. The olive tree is thought to have been domesticated in 7000 B.C in the Mediterranean region [134]. Cultivated olive *(Olea europaea* subsp. *europaea* var. *europaea*) is a woody fruit crop of major economic and social importance. The total olive cultivation area amounts to approximately 10.4 million hectares (mha) worldwide. In the Mediterranean region alone, olive cultivation occupies 9 mha, accounting for 80% of the global table and 98% of global olive oil production [135].

Well adapted to the semi-arid climate of the Mediterranean, olives have been historically planted in low-density systems in rainfed environments; nevertheless, the increasing demand for olive products has led to intensive planting under irrigation systems [136]. In recent years, olive productivity has been threatened by the ever-growing frequency and intensity of abiotic stressors experienced in the Mediterranean basin [137]. In this respect, olive yields were severely affected by drought and extreme weather events in the south of the EU throughout 2022 and the spring and summer of 2023. As a result, the production of olive oil dropped to its lowest level since 1994–1995, and market prices escalated (https://worldpopulationreview.com, accessed on 15 December 2024). Model climate forecasts indicate that intensified warming and drought are anticipated in many parts of the planet in the coming decades. According to these projections, heat and drought conditions will deteriorate significantly in areas already facing climate risks. In this context, olive trees will encounter severe climate pressures due to the increased temperatures and altered precipitation patterns anticipated in the Mediterranean region [96,135]. In addition, environmental changes are expected to exacerbate susceptibility to disease and have severe effects on olive production [135]. Recently, olive-grove-management systems have focused on incorporating sustainable agroecological practices, including effective soil management, composting, and the reutilization of waste products, to cope with the negative consequences of climate pressures [138]. In addition, exploring olive genetic variability, genetic/epigenetic mechanisms, and olive–microbiota interactions related to abiotic stress responsiveness is an alternative approach of great promise and is discussed below.

### 3.2. The Genetic and Epigenetic Component

Multiple studies have attempted to investigate the genetic/molecular basis of abiotic stress responses by employing molecular markers and a suite of multi-omics tools across a wide range of olive genotypes [139]. SSR and SNP markers have been used extensively to discriminate among different varieties and link distinct genotypes to crucial agronomic traits [140]. Recently, the most complete genomic variation map based on 89 olive tree genotypes was reported [141], providing a valuable tool for illuminating the genetic diversity among varieties as well as the molecular basis of fruit quality and stress response traits.

Transcriptomic studies using a salt-tolerant variety (Kalamon) and a salt-sensitive variety (Chondrolia Chalkidikis) subjected to salt stress demonstrated differential gene expression between the two genotypes and suggested that the transcription factors of the JERF and bZIP families are putative modulators of the salt-responsive gene regulatory networks [95]. Similarly, comparative morpho-physiological and transcriptomic studies under high-salt conditions revealed marked upregulation of the genes *OeNHX7*, *OeP5CS*, *OeRD19A,* and *OePetD* in a salt-tolerant variety (Royal de Cazorla) compared with medium-tolerance and susceptible varieties, indicating their key role in salt-tolerance mechanisms [142].

A meta-analysis of twenty-six RNA-Seq samples from *Olea europaea* and other fruit tree species identified sets of genes commonly regulated in drought stress conditions most likely involving transmembrane transport and metal ion binding or differentially regulated sets related to abscisic acid, gibberellin, brassinosteroids, and ethylene-activated signaling [94]. Notably, an Olive Atlas including 70 RNA-seq experiments spanning developmental processes and the response to a range of abiotic and biotic stresses, has been reported recently [143], constituting a valuable tool for analyzing gene networks and metabolic pathways implicated in stress response and tolerance.

Little has been reported on the epigenetic factors associated with abiotic stress mechanisms. Differential DNA methylation patterns were observed under high-salinity conditions among varieties with variable salt tolerance. Further analysis of the differentially methylated regions identified a set of underlying genes encoding OePIP1.1 (an aquaporin), OePetD (a cytochrome b6), OePI4Kg4 (a phosphatidylinositol 4-kinase), and OeXylA (a xylose isomerase), suggesting their implication and regulation by epigenetic mechanisms in the response to salt stress [96].

Overall, unraveling the molecular mechanism of abiotic stress responsiveness at the genetic and epigenetic level is crucial for comprehending the regulatory networks and cellular signaling pathways linked to abiotic stress tolerance in olive and will have significant ramifications for climate-resilient oliviculture.

### 3.3. The Microbiota Component

A series of studies have explored the effects of microbiota such as plant growth-promoting bacteria (PGPB) and plant growth-promoting fungi (PGPF) on olive’s tolerance to a variety of abiotic stressors in greenhouse and field conditions [144] (Table 2).

The PGPB used in these studies were sourced from the soil or rhizosphere of olive or other species as well as from PGPB collections. For example, Dias et al. explored the effects of the bacterium *Pseudomonas reactans* Ph3R3 in potted *Olea europaea* L. plants cv. Arbequina subjected to water deficiency conditions. It was demonstrated that Ph3R3 treatment enhanced plant performance by reducing water loss and improving N levels, the net CO_2_ assimilation rate, and the antioxidant capacity [123]. Similarly, the inoculation of cv. Arbequina with PGPB *Bacillus G7* protected plants from high-salt stress. *Bacillus G7* treatment improved physiological and metabolic parameters by increasing photosynthetic capacity, net carbon fixation, water use efficiency, and the accumulation of osmolytes and antioxidant molecules [145]. Transcriptional reprogramming was also evidenced, involving the induction of ABA pathway- and ion homeostasis-related genes. In another study, PGPB consortia sampled from the soil and rhizosphere of Tunisian olive orchards were used to inoculate drought-susceptible and drought-tolerant potted olive plantlets that were subsequently exposed to water deficit stress. The PGPB consortia were able to confer drought tolerance to olive plants from both cultivars [124]. Following a different approach, Sallami et al. determined microbial profiles from olive rhizosphere samplings across arid and semi-arid regions of Morocco and assessed plant growth bacterial performance under various conditions [146]. A large proportion of the isolates displayed marked tolerance to high-salinity conditions, highlighting their potential as candidates for bioinoculant formulations.

Concerning the effect of beneficial fungi, several studies have shown the positive impact of AMF on olive trees under abiotic stress exposure. The inoculation of potted olive plants with AMF (*Rhizophagus irregularis*) reinforced the tolerance to water deficit. This was attributed to enhanced olive plant growth, improved water status, the increased accumulation of osmolytes and antioxidants, and the modulation of phytohormones [125,126].

Furthermore, certain AMF mixtures confer tolerance to high-salt-stress conditions. Notably, inoculating young potted olive plants cv. Chetoui (salt-sensitive) with a mixture of *Glomus deserticola* and *Gigaspora margarita* alleviated the stress imposed by irrigation with salt-enriched wastewater. This has important implications for certain Mediterranean agricultural regions where the reuse of treated wastewater, often rich in salts, constitutes common practice [127].

Thus far, information regarding the molecular mechanisms and potential implication of epigenetic factors in the olive-tree-associated microbiome interactions is obscure. On the other hand, noteworthy research has been conducted to characterize olive microbiomes and determine the genotype and environmental drivers that may shape microbial community composition and diversity [147,148,149,150]. Further investigations and a deeper understanding of the molecular pathways governing the interplay between olive trees and microbiota during abiotic stress will help dissect stress tolerance from the perspective of the holobiont and contribute to alternative strategies for protecting oliviculture against climate challenges.

## 4. Other Woody Fruit Crops

### 4.1. Date Palm

Date palm (*Phoenix dactylifera* L.) is a socioeconomically significant crop throughout the Middle East and North Africa, contributing to food security in semi-arid and arid regions, where it remains the main woody plant. The date palm can grow in a wide range of climates, from Australia to Asia, Africa, and the Americas. Since it is acclimated to a wide range of temperatures (12.7 to 27.5 °C on average), it can tolerate both frost and very high temperatures of −5 and +50 °C [151] together with prolonged water shortage [152]. Although *P. dactylifera* can withstand drought and salt stress, intense water scarcity and ever-rising soil salinity have jeopardized the crop’s productivity. Specifically, the date palm suffers from high soil salinity despite being a relatively salt-tolerant plant. Consequently, *P. dactylifera* is a useful model for analyzing the physiological and molecular mechanisms that enable plants to withstand harsh weather conditions [153]. Studies over the last several years on date palm have begun to elucidate the physiological mechanisms of abiotic stress tolerance and the genes and biochemical pathways that control the response to these stresses.

#### 4.1.1. Genetic/Epigenetic Factors in Abiotic Stress

Recent reviews have reported on the genetic and genomic advancements made in date palm for facilitating targeted crop improvement strategies, such as abiotic tolerance in harsh environments [154,155]. Several of these studies have focused on transcriptomic analyses to unravel the genes and mechanisms that contribute to the development of salinity tolerance [155,156]. Using transcriptome and metabolomic profiling, Safronov et al. (2017) [157] investigated the adaptation methods of date palms under mild heat, drought, and combined heat and drought. In all three situations (drought, heat, and combined heat and drought), transcriptome data revealed the transcriptional activation of genes linked to reactive oxygen species, indicating the enhanced activity of enzymatic antioxidant systems in the cytosol, chloroplast, and peroxisome. Of note, there was a considerable enrichment for circadian and diurnal rhythm motifs in the genes that were differentially expressed under heat and combined heat and drought conditions, indicating new stress avoidance methods. For instance, since certain genes encoding heat shock factors (HSP) harbor circadian control motifs in their promoter regions, it is conceivable that the synthesis of HSP is synchronized with the time of the day as required by the circadian clock. Another transcriptome study identified genes implicated in detoxifying cadmium (Cd) toxicity [158]. Numerous defense- and detoxification-related genes, including those encoding heavy metal (HM)-chelators and HM-transporters, were successfully predicted in response to Cd stress. This provided a strong basis for the study of the molecular regulation mechanisms of heavy metal accumulation and tolerance in date palm leaves and roots. The primary and secondary metabolic profiles, along with memory effects on water relations, appear to prime date palm foliar characteristics for recurrent summer drought occurrences, according to a study of the metabolic profile of date palm seedlings subjected to a drought-recovery regime in both summer- and winter-simulated climates [159]. Date palms that have a well-coordinated metabolic network—which includes the antioxidative system, the accumulation of appropriate solutes, osmotic adjustment, and cell-membrane stability—seem to be less vulnerable to drought. These drought-compensating strategies may be required more frequently during the summer. Xu et al. demonstrated the implication of *salt overly sensitive* (*SOS*) pathway genes, including *PdSOS2;1*, *PdSOS2;2*, *PdSOS4*, *PdSOS5*, and *PdCIPK11*, in the salt response [99]. The gene expression was in line with the changes in physiological parameters such as amino acid profile and Na^+^/K^+^ homeostasis, which in turn inhibited plant growth. The “spliceosome” pathway was enriched in the upregulation category, suggesting that alternative splicing (AS) may play a role in the date palm’s response to salt stress.

Climate chamber experiments and proteomic analysis provided a complete view of how the date palm leaf proteome may adapt to Saudi Arabia’s natural environment [97]. It appears that the date palm has evolved a complex multi-mechanism based on increasing abundances of heat shock proteins involved in abiotic stress defense as well as redox homeostasis proteins and proteins involved in isoprene production to counteract the stress imposed by summer temperature conditions and the soil aridity of the Arabian Peninsula.

Mueller et al. [98] used integrative multi-omics studies, followed by focused metabolomics, hormone, and ion investigations, after exposing date palm to a salt stress dose equal to seawater for up to four weeks. A strikingly high degree of convergence between gene expression and protein abundance was discovered when the proteomic data were integrated with the transcriptomic data. This clarifies the mechanisms of acclimatization that are used, which rely on reprogramming protein production. Date palm successfully combines several salt-tolerance mechanisms present in both halophytes and glycophytes for growth in highly saline environments: “acclimation” through osmotic adjustment, reactive oxygen species scavenging in leaves, and remodeling of the ribosome-associated proteome in salt-exposed root cells, as well as “avoidance” through effective sodium and chloride exclusion at the roots.

Further attempts to identify and functionally characterize salt- and drought-tolerance-related genes in date palm have been made by several research groups [131,157,160]. Due to the lengthy regeneration time, the complexity of the palm genome, and lack of efficient regeneration protocols, genetic engineering and GE techniques have not yet been effectively applied in date palm breeding, despite their immense value [20,161]. Similarly, recent GWASs and genomic prediction studies [24,162,163] have provided valuable insights into the genetic factors influencing date palm fruit traits; nevertheless, GWASs on genes or quantitative trait loci associated with abiotic and biotic stress tolerance have not been reported thus far.

The role of epigenetic mechanisms in date palm gene regulation under abiotic stresses has not yet been thoroughly studied, despite its significance. Comparative miRNA profiling under salt stress showed a differential expression of miRNAs in leaf and root tissue, whereas most miRNA sequences were upregulated in both tissue types by salinity treatment [160]. Al-Harrasi et al. investigated the DNA methylation status and transcriptome profile in date palm under salt stress. Whole-genome bisulfite sequencing (WGBS) revealed differential DNA methylation in the roots of date palm plants upon salinity treatment [100]. Gene expression was impacted by 5mC methylation alterations in different gene regions; however, DNA methylation was not the only factor affecting global transcript abundance. These findings emphasize the significance of DNA methylation in stress-induced epigenetic remodeling and may be useful for crop breeding programs.

#### 4.1.2. Microbiota Aspects and Abiotic Stress

Microbial communities in oasis habitats enable plants to withstand harsh environmental conditions, which is where *Phoenix dactylifera* flourishes [164]. Even though endophytic bacteria promote plant growth in the face of abiotic stress [107], little is known about *P. dactylifera* endophytic bacteria and how they contribute to the development of abiotic tolerance, including drought and salt tolerance.

Date palm roots choose a variety of endophytic communities that can support plant growth in drought-prone environments, according to a study by Cherif et al. on the ecology of date palm root endophytes from oasis desert farms in southern Tunisia [128]. In another study, the morphological and growth characteristics of date palm seedlings were negatively impacted by prolonged exposure to salt stress. Because the rhizosphere is the primary source of endophytes, changes in rhizosphere populations are likely to directly affect the makeup of the endophytic community. Yaish et al. (2016) identified endophytic bacterial and fungal communities in *P. dactylifera* grown under salt stress and showed that the composition of these microbial communities changed significantly in response to changes in salinity [165].

Bacterial strains isolated from the rhizosphere of date palms cultivated in an orchard with high soil salinity were examined for their ability to promote plant growth through the generation of microbial volatile compounds (mVOCs) [131]. The strain ‘*Enterobacter cloacae* SQU-2 (SQU-2)’ was found to produce mVOCs, which improved the growth of *Arabidopsis thaliana* Columbia (Col-0) seedlings under both normal and salt stress conditions. Inspection of the rhizobacterium’s genome using next-generation sequencing techniques demonstrated the existence of mVOC gene clusters, suggesting the function of mVOC synthesis pathways in the growth enhancement of date palm plants confronted with high-salinity stress. These findings encourage additional research on the mechanisms underlying the plant–microbe interaction that promotes development under unfavorable conditions and its potential use in agriculture. Sabeem et al., 2022 reported that the root colonization of date palm seedlings with the beneficial endophyte *Piriformospora indica* significantly reduced the detrimental effects of salt stress through enhanced growth through ion homeostasis and nutrient uptake, antioxidant activity, and the modulation of stress-responsive genes [129]. Ensuing studies of root transcriptomes demonstrated that *P. indica* colonization resulted in the upregulation of multiple genes involved in metabolic and signaling pathways relevant to salt tolerance [130]. Date palm root colonization by *P. indica* is a remarkable example of a beneficial microbial symbiosis. Presently, the underlying molecular mechanisms by which it establishes itself and exerts its beneficial effects are poorly understood and are anticipated to be the focus of rigorous future research.

A comprehensive review by Du et al., 2025 [166] highlights the diverse defense mechanisms enabling date palms to thrive in deserts. Beyond common strategies like avoidance, osmotic adjustments, and ROS scavenging, date palms utilize efficient root-to-shoot communication, even with limited xylem transport. Heat shock proteins aid temperature tolerance, whereas unique root adaptations enhance resistance to ozone, drought, and salinity. Their findings suggest that single-strategy approaches are ineffective in extreme environments, underscoring the importance of understanding the interplay of different mechanisms for future research on date palm resilience.

### 4.2. Pomegranate

1. Pomegranate (*Punica granatum* L.) is an ancient perennial species native to Central Asia and has been cultivated for over 3000 years. Today, it is commercially grown in more than 30 countries, including India, Iran, Spain, China, and the United States. It is also a major subtropical fruit crop in the Mediterranean region, grown especially in Turkey and Spain, and is valued for its fruits, leaves, and other plant parts due to its antioxidant properties. The tree thrives in arid and semi-arid regions, often facing challenges such as salinity and other environmental stressors, which can negatively impact transplantation survival rates, fruit yield, and quality [167,168].
2. However, pomegranate trees thrive in warm climates and have a low tolerance for cold temperatures, leading to their predominant cultivation in tropical and subtropical regions. Cold stress restricts plant growth, development, and yield, with sudden cold snaps in winter and late spring posing a particular threat to pomegranate trees. These temperature drops can cause freezing damage, significantly reducing fruit yield and quality, thereby affecting market availability [169].

#### 4.2.1. Genetic Aspects and Transcriptional Regulation

Gene expression studies have revealed numerous gene sets involved in a variety of metabolic, antioxidant, and signaling pathways during pomegranate exposure to abiotic stress factors. Sugars play an active role in the regulation of growth, photosynthesis, carbon partitioning, carbohydrate and lipid metabolism, osmotic homeostasis, protein synthesis, and gene expression during various abiotic stresses. The concentration of sugars determines the expression of these sugar-regulated genes, especially those involved in photosynthesis, sucrose metabolism, and osmolyte synthesis. An increase in the concentration of soluble sugars, such as glucose, sucrose, and fructose, enhances plant tolerance to several abiotic stresses, such as drought, salinity, and cold [170,171]. To cope with different abiotic constraints, plants tightly regulate the vacuolar storage and transport of sugars. Sugar transporters across cell membranes mediate sugar translocation. In plants, the most recent type of sugar transporters, the Sugar Will Eventually Be Exported Transporters (*SWEET*) gene family, which includes hexose and sucrose transporters, is widely distributed and functions as uniporters, facilitating the passive transport of sugar molecules across cell membranes. SWEET is involved in various physiological processes, including phloem loading, senescence, pollen nutrition, grain filling, nectar secretion, and the regulation of both abiotic stresses (such as drought, heat, cold, and salinity) and biotic stress responses [172,173].

Earlier studies in cotton and tomato demonstrated the role of *SWEET* genes in responding to various abiotic stresses, such as salt, heat, and cold stress, by regulating the redistribution of soluble sugars. This process helps maintain osmotic balance and supports plant growth under challenging environmental conditions [174,175]. Kumawat et al. [101] identified and characterized 15 *SWEET* genes in the pomegranate genome. Their chromosome distribution, exon/intron structure, phylogenetic relationships, conserved motifs, and expression dynamics during fruit development were analyzed. RNA-seq data analysis and qPCR results confirmed the tissue-specific expression of candidate *SWEET* genes. Furthermore, the identification of differentially expressed *SWEET* genes under high-salinity stress provides valuable insights into the sugar transport mechanisms across different tissues in response to abiotic stress. The findings of this study contribute to fundamental knowledge of *PgSWEET* genes and will support future research in understanding their role in pomegranate.

Several studies have investigated the genetic basis of salt stress responses to uncover the molecular and physiological strategies that pomegranate plants employ to adapt and survive in high-salt environments. To support this goal, whole-genome sequencing and de novo assembly have been performed on several commercially important cultivated varieties of pomegranate—such as the main Indian cultivar ‘Bhagawa’, the elite cultivar ‘Dabenzi’, and ‘Taishanhong’, a widely grown cultivar in China. The genome sequence of pomegranate provides a valuable resource for dissecting various biological and biochemical traits and offers important insights for accelerating breeding efforts [176,177,178]. In addition, Patankar et al. [179] presented a high-quality chromosome-level reference genome that will aid in identifying stress resistance traits in pomegranates growing in arid environments. The final assembly reached 384.65 Mb, with an N50 contig size of 43.11 Mb, and 353.42 Mb of the genome was anchored onto eight pseudo-chromosomes. Genome annotation revealed that 48.79% of the genome consists of repetitive elements and includes 21,620 protein-coding genes.

A high-quality, chromosome-level reference genome for the ‘Sanbai’ pomegranate variety, known for its resistance to freezing temperatures, has been successfully delivered, with a remarkable contig N50 of 15.93 Mb. This comprehensive genome assembly, improved by long-read sequencing across 38 pomegranate accessions, led to the discovery of 14,239 polymorphic structural variants, highlighting their essential roles in genomic diversity and population differentiation related to cold tolerance. Notably, a significant 5.4 Mb inversion on chromosome 1 was identified, which played a key role in the plant’s cold resistance. Additionally, by combining bulked segregant analysis (BSA), differential selection analysis, and genetic transformation techniques, the interaction between the PgNAC12 transcription factor and PgCBF1 was identified and confirmed, demonstrating their crucial roles in the response to cold stress. These findings represent a major step forward in pomegranate genomics, providing new insights into the genetic mechanisms of cold tolerance and offering valuable resources for improving soft-seeded pomegranate varieties through genetic improvement [180].

Liu et al. [102] conducted deep transcriptome sequencing of pomegranate roots and leaves under salinity stress, revealing extensive differential gene expression, with 1080 upregulated genes and 1175 downregulated genes. Most differentially expressed genes (DEGs) exhibited tissue- and time-dependent transcript accumulation. In the roots, the genes involved in cell wall organization, transmembrane transport, and oxidation–reduction processes were downregulated, whereas those related to proteolysis and metabolic activities were upregulated. Conversely, in leaves, 41.29% of DEGs were initially suppressed but later recovered, including genes linked to ion and metal ion binding. The ion transport and oxidation–reduction processes were notably restricted. In addition, several DEGs were associated with abscisic acid (ABA)- and calcium (Ca^2+^)-related pathways, as well as mitogen-activated protein kinase (MAPK) signaling cascades, including ABA receptors, Ca^2+^ sensors, MAPK components, and transcription factors. Furthermore, functional genes encoding heat shock proteins (HSPs), late embryogenesis abundant proteins (LEAs), aquaporins (AQPs), and peroxidases (PODs) were identified. This study enhances our understanding of the molecular mechanisms underlying pomegranate’s response to salinity and provides a foundation for identifying salt-tolerant genes to support breeding efforts.

Similarly, Tang et al. [103] used the ‘Taishanhong’ variety to explore the response mechanisms of pomegranate leaves to salt stress. Their time-course experiment on pomegranate seedlings exposed to different salt concentrations demonstrated notable changes in physiological traits and gene expression. The induction of proline, total soluble sugar, and superoxide dismutase and peroxidase (SOD/POD) activities was accompanied by differential gene expression 72 h after salinity stress. In total, 6571 differentially expressed genes (DEGs) were identified across treatments, including 374 transcription factors (TFs), such as AP2/ERF, bHLH, MYB, and WRKY, suggesting their potential roles in regulating salt stress responses in pomegranate. Weighted gene co-expression network analysis (WGCNA) uncovered 6 distinct modules and 180 hub genes strongly associated with salt stress. Functional annotation further highlighted primary and secondary metabolism, as well as key signaling pathways, as major contributors to stress adaptation. These findings provide valuable insights into the molecular basis of salt tolerance in pomegranate leaves, offering a theoretical framework for improving plant salt tolerance through genetic engineering.

Furthermore, several studies have explored the effect of abscisic acid (ABA), a common phytohormone, which regulates various physiological processes ranging from stomatal opening to protein storage and provides adaptation to many stresses like drought, salt, and cold. ABA also acts as a signaling mediator for regulating the adaptive response of plants to different environmental stress conditions [181].

Morpho-physiological analyses and transcriptome profiling of pomegranate under drought stress revealed that the application of exogenous ABA significantly improved drought tolerance [168]. In this study, pomegranate cuttings were exposed to drought stress for 90 days while being treated with ABA at three different concentrations (30, 60, and 90 μM). The results indicated that compared with untreated plants, ABA enhanced pomegranate growth and related physiological responses. Additionally, a comparative transcriptome analysis between ABA-treated and untreated pomegranates uncovered the mechanisms triggered by ABA in response to drought stress. The findings demonstrated that exogenous ABA application strengthened key metabolic pathways, including brassinosteroid synthesis, peroxisome biogenesis, photosynthesis, and hemicellulose synthesis, thereby improving drought resistance. However, excessive ABA application activated its degradation process and a regulatory feedback loop in pomegranate to control ABA accumulation beyond the optimal level. This, in turn, led to inhibited growth and reduced stress resistance.

Although grown in warm environments, pomegranate may face cold spells in winter and early spring with catastrophic consequences. Plants have developed complex biochemical and physiological mechanisms and a flexible transcriptional network regulated by a series of transcription factors to adapt to cold stress. Genes encoding C-repeat binding factors (CBFs) are known to have an important function in plants’ cold resistance [182,183]. Following exposure to 4 °C, two *CBF* genes (*PgCBF3* and *PgCBF7*) exhibited significantly upregulated expression in the cold-tolerant *Punica granatum* ‘Yudazi’ compared with the cold-sensitive ‘Tunisia’. *PgCBF3* was localized in the nucleus, while *PgCBF7* was present in the cell membrane, cytoplasm, and nucleus, both demonstrating transcriptional activation activity in yeast. A yeast one-hybrid assay and a dual-luciferase reporter assay confirmed that *PgICE1* (*Inducer of CBF Expression*) specifically binds to and significantly enhances the promoter activity of *PgCBF3* and *PgCBF7*. Compared with wild-type plants, transgenic *Arabidopsis thaliana* lines expressing these genes exhibited higher survival rates following cold treatment. These plants also showed increased levels of soluble sugar and proline, along with reduced electrolyte leakage, malondialdehyde content, and reactive oxygen species (ROS) production. Additionally, they displayed an enhanced enzymatic activity of catalase, peroxidase, and superoxide dismutase while upregulating the expression of *AtCOR15A*, *AtCOR47*, *AtRD29A*, and *AtKIN1*. Overall, *PgCBFs* were positively regulated by PgICE1, which subsequently mediated the expression of downstream cold-regulated (*COR*) genes, thereby enhancing freezing tolerance [104].

In a similar study, calmodulin-binding transcriptional activator (CAMTA) family proteins, which are key regulators of cold stress tolerance in plants, were studied across 12 species, including pomegranate [184]. Pomegranate *CAMTA3* (*PgCAMTA3*) was isolated and characterized, and it demonstrated enhanced cold tolerance when expressed in *Arabidopsis thaliana*. Quantitative real-time PCR (qRT-PCR) analysis showed that the expression of *PgCAMTA3* was upregulated under cold and ABA treatments. The overexpression of *PgCAMTA3* in *A. thaliana* transgenic lines enhanced cold stress tolerance. A. *thaliana* transgenic plants exhibited higher survival rates under cold stress and higher enzymatic activity, e.g., peroxidase (POD), catalase (CAT), and superoxide dismutase (SOD). These antioxidant enzymatic activities collectively contribute to better cold stress tolerance by providing more effective reactive oxygen species (ROS) scavenging and cellular protection mechanisms, which was confirmed by lower levels of malondialdehyde (MDA) and ROS production. In addition, the overexpression of *PgCAMTA3* led to the upregulation of the expression levels of *AtCBF2*, *AtNCED3*, and *AtWRKY22*, which were modulated by *CAMTA3.*

Furthermore, Guan et al. [105] investigated the physiological changes and transcriptome profiles of Tunisian soft-seed pomegranate under cold (6 °C) and freezing (0 °C) stress. The study compared two groups, 6 °C vs. CK (normal-temperature control) and 0 °C vs. CK, identifying a large number of DEGs. A total of 1561 genes were commonly expressed in both comparisons, indicating their role in the plant’s response to cold and freezing stress. Additionally, the researchers identified 6211 genes exclusively expressed under cold stress and 1685 genes specific to freezing stress. DEGs under cold stress were primarily associated with transcription factors (TFs), including the bHLH, NAC, and ERF families. The other affected pathways included photosynthesis, osmotic regulation, and hormone signal transduction. Potential cold response/resistance genes were also identified, particularly those involved in plant hormone signaling, photosynthesis, carbon fixation in the C4 pathway, and sucrose and galactose metabolism. Physiological analyses further confirmed that both cold and freezing stress caused membrane damage as well as the accumulation of soluble sugar, soluble protein, and proline. Moreover, photoinhibition occurred due to damage to the photosynthetic apparatus, leading to a decline in the light energy conversion efficiency and electron transfer rate. These effects were significantly more severe under freezing stress. Overall, this study provides valuable insights into the molecular mechanisms of pomegranate’s cold stress response, laying a foundation for the identification of key candidate genes for molecular breeding to enhance cold tolerance in pomegranate.

Other studies have investigated the involvement of Beta-amylases (BAMs), a protein family known to have an important role in breaking down starch into soluble sugars, contributing significantly to sugar accumulation during cold stress. These soluble sugars are then translocated from the chloroplasts to the cytoplasm, where they participate in energy metabolism, strengthening the plant’s defense against cold-induced damage [106,185]. Liu et al. [106] conducted genome-wide analyses on eight *PgBAM* genes from the pomegranate genome dataset to explore their role in cold stress tolerance. These genes were unevenly distributed across chromosomes and were categorized into four groups based on their orthologous members. A syntenic analysis revealed that *PgBAM1*, *PgBAM4*, and *PgBAM5* shared relationships with *BAM* genes from *Arabidopsis*, *kiwifruit*, and *Chinese white pear*.

Promoter binding motif prediction suggested that *PgBAM* genes may play roles in the light response, stress adaptation, hormone signaling, and developmental processes. Gene expression indicated that *PgBAM4* was predominantly expressed in leaves, *PgBAM7* in flowers, and *PgBAM8* in roots, leaves, and fruit during ripening, particularly in the pericarp. Transcriptome analysis of the cold-sensitive cultivar ‘Tunisia’ seedlings subjected to cold stress for 0 and 12 h identified the starch and sucrose metabolism pathway as key to the cold stress response in pomegranate seedlings. *PgBAM4* showed strong expression and was linked to cold-responsive *BAM* genes, correlating with soluble sugar levels and cold stress resistance in ‘Tunisia’ (cold-sensitive) and ‘Sanbai’ (cold-tolerant) seedlings. Moreover, yeast one-hybrid assays confirmed that PgCBF7, a transcription factor for freezing tolerance, binds to the *PgBAM4* promoter, underscoring its regulatory role in cold adaptation.

#### 4.2.2. Microbiome and Abiotic Stress

Few studies have been reported thus far concerning pomegranate microbiomes and plant–microbe interactions under abiotic stress conditions. Bompadre et al. [132] investigated the impact of inoculating two strains of arbuscular mycorrhizal fungi (AMF), *Rhizophagus intraradices* (N.C. Schenck & G.S. Smith) C. Walker & A. Schüßler (GA5 and GC2), on pomegranate plants grown under two different irrigation regimes. The plants’ responses to oxidative stress varied depending on the type of tissue and the severity of the stress. Their results indicated that mycorrhizal plants enhanced their antioxidant defenses—specifically the ROS-scavenging enzymes superoxide dismutase (SOD), catalase (CAT), and ascorbate peroxidase (APX)—in shoots across both irrigation treatments, whereas the root response was inconsistent. AMF inoculation helped regulate malondialdehyde (MDA) levels, possibly by swiftly boosting antioxidant defenses and preventing lipid peroxidation. The study concluded that early inoculation with AMF, especially for the GC2 strain, offers protection to pomegranate plants against abiotic stress during propagation.

Characterizing the microbiota communities’ diversity and structure in different conditions has begun to be reported. For instance, Ravinath et al. [186] utilized 16S rRNA amplicon-based metagenomics to identify the dominant and abundant bacterial species in the rhizosphere of the pomegranate ‘Bhagawa’ variety at various soil depths. Considering that beneficial microorganisms in the rhizosphere play a functional role in plant protection and resilience to abiotic factors, future research efforts are expected to focus on the pomegranate microbiome and pomegranate–microbe interactions.

## 5. Conclusions and Future Perspectives

Climate change and the exponential growth of the human population raise major concerns for crop production, sustainable agriculture, and food security. Abiotic stress pressures, such as increased drought, elevated temperatures, extreme salinity, and unpredictable rainfall patterns, negatively affect plant development, yield, and quality.

Plants have evolved complex mechanisms to counteract stress and adapt to changing environments.

Molecular mechanisms operating at the genetic and epigenetic level, engaging multiple gene networks and metabolic pathways as well as the intricate interactions between the plant and its microbial communities, have been shown to play crucial roles in abiotic stress responsiveness and tolerance. Most studies involved in understanding the molecular basis of abiotic stress tolerance have been performed on annual herbaceous species, including important crops such as tomato, legumes, and cereals. In recent years, the molecular mechanisms underlying abiotic stress responses have begun to be characterized in key woody fruit crops of high economic value cultivated in the Mediterranean region, such as grapevine, olive, date palm, and pomegranate.

The Mediterranean basin experiences enhanced climatic pressures, whereas climate forecasts predict increased water scarcity and warming conditions for the decades ahead, jeopardizing the viability of key woody perennial fruit crop cultivation. Further research is needed to address environmental threats and develop ways to impart resilience while sustaining good yields and fruit quality. Insights into abiotic stress responses at the genetic and epigenetic level and the host plant–microbiota interaction will greatly contribute to a deeper understanding of the molecular processes related to stress tolerance. Subsequently, this will facilitate the development of biomarkers linking genotypes to stress-relevant traits, discriminating among cultivars with different susceptibility/resilience and selecting suitable genotypes in breeding programs, ultimately leading to the development of stress-tolerant varieties. Further research should focus on additional and more detailed investigations into the molecular mechanisms underlying abiotic stress tolerance through the synergistic use of molecular tools such as -omics, GWASs, and genome editing. Genome editing methods like CRISPR/Cas offer exciting new opportunities for precise genetic/epigenetic modification, targeting genomic regions with abiotic stress relevance [187]. CRISPR/Cas can expedite crop improvement as it circumvents the long and cumbersome cycles of the breeding process, especially in woody perennial species. Moreover, rapid advancements in the CRISPR/Cas field concerning transgene-free systems make this biotechnology particularly attractive. Nevertheless, the low regeneration capacity of genome-edited plants and the regulatory framework limitations hinder the effective implementation of genome-editing technology [21,188]. In this context, more progress is needed in this domain toward efficient transformation and regeneration systems across different genotypes as well as easing regulations and harmonizing the legal framework among countries.

Finally, characterizing woody crop microbiomes under different conditions, determining the genotype and environmental drivers of microbial community structure/function, and exploring the potential of beneficial microbiota to confer tolerance are of major importance. Elucidating the role of host plant–microbiota interaction in stress protection will allow for embracing new perspectives in crop improvement, such as the emerging concept of microbiota-assisted breeding where the holobiont is considered a breeding target and the microbiome is considered a trait [189]. In addition, it could result in the development of effective bio-inoculations with suitable consortium cocktails, enabling crops to alleviate the damaging effects of stress and withstand environmental pressures in the field.

Overall, the research endeavors and outcomes mentioned above will lead to effective synergistic strategies to tackle climate pressures, enhance fitness and adaptability, and safeguard sustainable fruit tree cultivation under unfavorable environmental conditions.

Further insights into the intertwined genetic, epigenetic, and microbial mechanisms underlying abiotic stress tolerance will unravel the complex ‘crop-microbiome-stress’ network and ultimately enhance the resilience of key woody perennial fruit crops in an era of water scarcity and global warming.
